# HIV-1-related cardiovascular disease is associated with chronic inflammation, frequent pericardial effusions and increased myocardial oedema

**DOI:** 10.1186/1532-429X-18-S1-O104

**Published:** 2016-01-27

**Authors:** Ntobeko A Ntusi, Eoin O'Dwyer, Lucy Dorrell, Stefan K Piechnik, Vanessa M Ferreira, Theodoros D Karamitsos, E Sam, Kieran Clarke, Stefan Neubauer, Cameron Holloway

**Affiliations:** 1 grid.1005.40000000449020432University of NSW, Darlinghurst, NSW Australia; 2 grid.4991.50000000419368948OCMR, University of Oxford, Oxford, UK; 3 grid.437825.f0000000091192677Cardiology, St. Vincent's Hospital Sydney, Sydney, NSW Australia; 4 grid.4991.50000000419368948University of Oxford, Oxford, UK

## Background

Treatment for HIV has been associated with a near-normal life expectancy, though with increased cardiac morbidity and mortality. Mechanisms of heart disease in patients infected with HIV remain unclear. Coronary artery disease and myocardial dysfunction may be partly related to untreated chronic inflammation. Cardiovascular magnetic resonance (CMR) imaging allows a comprehensive assessment of myocardial structure, function and tissue characterization. We investigated, using CMR, the presence of subclinical cardiac inflammation and myocardial disease in asymptomatic HIV-infected individuals.

## Methods

Myocardial structure and function were assessed using CMR at 1.5 Tesla in treated HIV-infected individuals without overt cardiovascular disease (n = 103, mean age 45 ± 10 years), compared to healthy controls (n = 92, mean age 44 ± 10 years). Assessments included left ventricular volumes, ejection fraction, strain, regional systolic, diastolic function, native T1 mapping, oedema and gadolinium enhancement. CMR imaging biomarkers were correlated with serum hs-CRP and markers of HIV severity.

## Results

Compared to controls, subjects with HIV infection had 4% lower left ventricular ejection fraction (< 0.001), 7% higher myocardial mass (p = 0.02) and 41% lower peak diastolic strain rate (p < 0.001). Compared to controls, subjects with HIV had 4% higher STIR values and 8% were above the set STIR threshold of 40 mm2. There were higher native T1 values in subjects with HIV (969 ms ± 28 versus 956 ± 24 in controls, p = 0.01,). There were greater numbers of subjects with HIV above a set T1 threshold of 990 ms, compared to controls (9% vs. 3%, p < 0.001). Pericardial effusions and myocardial fibrosis was three and four times more common, respectively, in subjects with HIV infection, compared to controls (both p < 0.001).

## Conclusions

Treated HIV infection is associated with changes in myocardial structure and function, in addition to higher rates of subclinical myocardial oedema and frequent pericardial effusions. Chronic systemic inflammation in HIV, which involves the myocardium and pericardium, may explain the high rate of myocardial fibrosis and increased cardiac dysfunction in people living with HIV.

**Figure 1:**
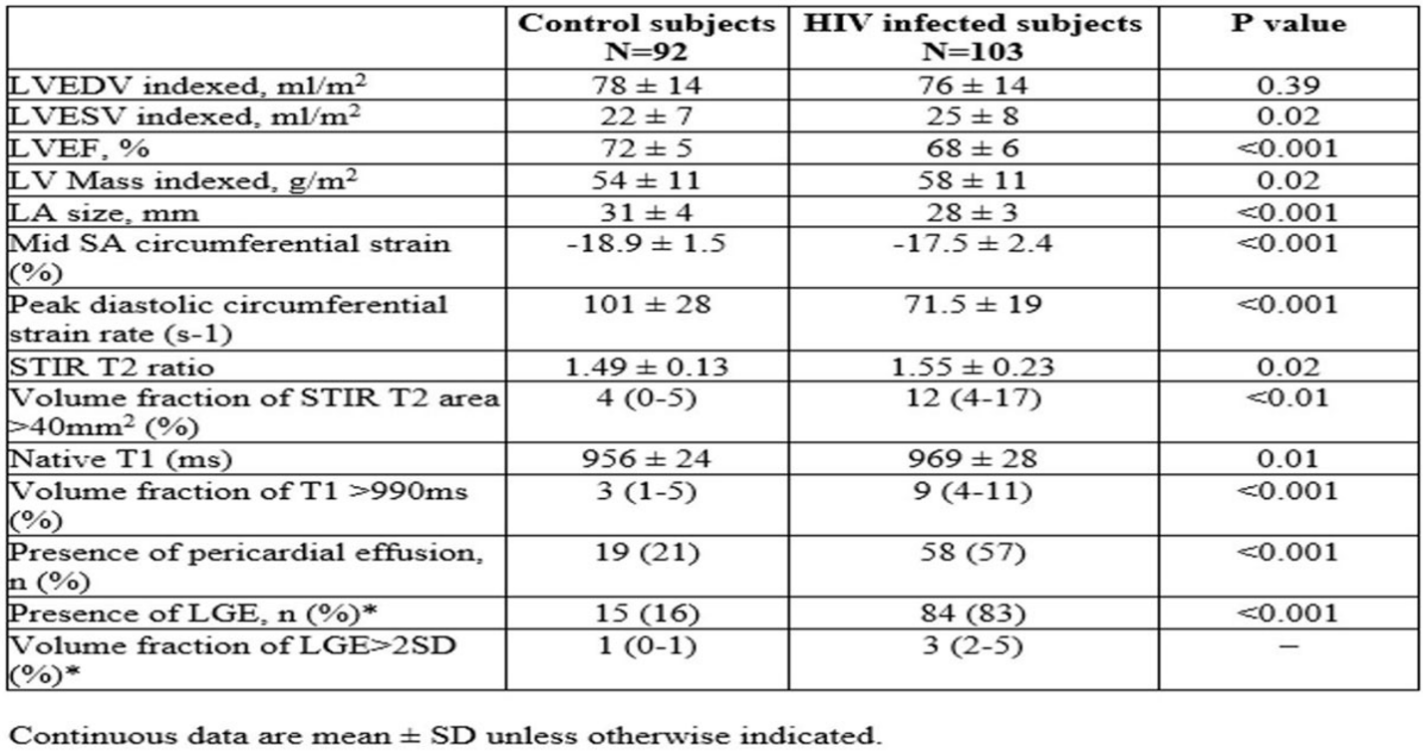
**CMR findings**.

